# Genome-wide identification and evolutionary analysis of the NRAMP gene family in the AC genomes of Brassica species

**DOI:** 10.1186/s12870-024-04981-1

**Published:** 2024-04-23

**Authors:** Yuquan Zhao, Qijun Xie, Qian Yang, Jiamin Cui, Wenqing Tan, Dawei Zhang, Jianhua Xiang, Lichao Deng, Yiming Guo, Mei Li, Lili Liu, Mingli Yan

**Affiliations:** 1 Hunan Key Laboratory of Economic Crops Genetic Improvement and Integrated Utilization, School of Life and Health Sciences, Hunan University of Science and Technology, Xiangtan, 411201 China; 2 Yuelushan Laboratory, Hongqi Road, Changsha, 410125 China; 3 Crop Research Institute, Hunan Academy of Agricultural Sciences, Changsha, 410125 China; 4 School of Life Science and Chemistry, Hunan University of Technology, Zhuzhou, 412007 China; 5 Hunan Engineering and Technology Research Center of Hybrid Rapeseed, Hunan Academy of Agricultural Sciences, Changsha, 410125 China

**Keywords:** *Brassica napus*, Brassica species, Bioinformatics, Cadmium, Subfamily, Motifs, Cis-acting element

## Abstract

**Background:**

*Brassica napus*, a hybrid resulting from the crossing of *Brassica rapa* and *Brassica oleracea*, is one of the most important oil crops. Despite its significance, *B. napus* productivity faces substantial challenges due to heavy metal stress, especially in response to cadmium (Cd), which poses a significant threat among heavy metals. Natural resistance-associated macrophage proteins (NRAMPs) play pivotal roles in Cd uptake and transport within plants. However, our understanding of the role of *BnNRAMPs* in *B. napus* is limited. Thus, this study aimed to conduct genome-wide identification and bioinformatics analysis of three Brassica species: *B. napus*, *B. rapa*, and *B. oleracea*.

**Results:**

A total of 37 *NRAMPs* were identified across the three Brassica species and classified into two distinct subfamilies based on evolutionary relationships. Conservative motif analysis revealed that motif 6 and motif 8 might significantly contribute to the differentiation between subfamily I and subfamily II within Brassica species. Evolutionary analyses and chromosome mapping revealed a reduction in the NRAMP gene family during *B. napus* evolutionary history, resulting in the loss of an orthologous gene derived from *BoNRAMP3.2*. Cis-acting element analysis suggested potential regulation of the NRAMP gene family by specific plant hormones, such as abscisic acid (ABA) and methyl jasmonate (MeJA). However, gene expression pattern analyses under hormonal or stress treatments indicated limited responsiveness of the NRAMP gene family to these treatments, warranting further experimental validation. Under Cd stress in *B. napus*, expression pattern analysis of the NRAMP gene family revealed a decrease in the expression levels of most *BnNRAMP* genes with increasing Cd concentrations. Notably, *BnNRAMP5.1/5.2* exhibited a unique response pattern, being stimulated at low Cd concentrations and inhibited at high Cd concentrations, suggesting potential response mechanisms distinct from those of other *NRAMP* genes.

**Conclusions:**

In summary, this study indicates complex molecular dynamics within the NRAMP gene family under Cd stress, suggesting potential applications in enhancing plant resilience, particularly against Cd. The findings also offer valuable insights for further understanding the functionality and regulatory mechanisms of the NRAMP gene family.

**Supplementary Information:**

The online version contains supplementary material available at 10.1186/s12870-024-04981-1.

## Background

Crops face the challenge of overcoming the adverse effects of abiotic stress, which can ultimately lead to decreased productivity [1, 2]. Cadmium (Cd), a highly toxic heavy metal, exerts its detrimental effects by binding to thiol groups in proteins, inhibiting enzyme activity, disrupting protein function, and interfering with the absorption of essential elements [3]. Consequently, these actions have profound implications for the physiological and biochemical functions of crops. Compounding this issue, cadmium readily accumulates in crops, posing a significant threat to human health through the food chain [4, 5]. Even low doses of Cd, when experienced through prolonged exposure, can have severe health implications [6]. Cadmium, classified as a nonessential element, lacks specialized transporters within plant systems. Its absorption primarily occurs through transporters designed for other metals, such as natural resistance-associated macrophage proteins (NRAMPs) [7]. NRAMPs, crucial proton/metal transporters in plants [8, 9], are involved in transporting various essential elements, such as zinc (Zn), iron (Fe), and manganese (Mn), as well as some nonessential elements, such as Cd or arsenic (As) [9, 10]. Consequently, NRAMPs play a pivotal role in maintaining metal homeostasis and detoxifying heavy metals in plant systems [11]. *NRAMPs* exhibit highly conserved domains and are widely distributed across genomes from bacteria to humans [12]. Extensive research on the NRAMP gene family has been conducted in plants such as *Arabidopsis thaliana* [13–15], *Oryza sativa* L [10, 16, 17]. , and *Medicago truncatula* [18].

NRAMP gene family transporters primarily facilitate the transport of divalent metal cations, such as Fe^2+^, Mn^2+^, and Cd^2+^, exhibiting variations in ion selectivity among different NRAMPs. For example, in *A. thaliana*, AtNRAMP1 is involved in high-affinity transport for Mn uptake in roots [15] and acts as a transporter for Fe [13]. Moreover, OsNRAMP1 exhibits wide affinity and is capable of transporting Fe, Cd, Mn, and As [9, 16]. Interestingly, rice OsNRAMP4 (also known as Nrat1) may enhance rice aluminum (Al) tolerance by reducing Al levels in the cell wall, where it is capable of transporting Al [18]. Unfortunately, the specific ion preferences and evolutionary relevance of NRAMP transporters have not been determined.

Gene expression patterns are intricately linked to physiological functions. For instance, AtNRAMP1 in *A. thaliana*, MtNRAMP1 in *M. truncatula*, MhNRAMP1 in *M. hupehensis* and OsNRAMP1 in *Oryza sativa* L. exhibit localization on the root plasma membrane [15, 18–20]. Under conditions of iron deficiency, *AtNRAMP1* transcripts accumulate primarily in roots and exhibit minimal accumulation in leaves [13]. Notably, *MtNRAMP1* exhibits the highest expression in both roots and nodules [18]. Overexpression of *MhNRAMP1* leads to increased transport of Cd from roots to leaves and heightens the susceptibility of yeast, tobacco, and apple callus tissues to Cd [19]. Additionally, the knockout of *OsNRAMP1* significantly diminishes the uptake of Cd and Mn in rice roots, subsequently impacting their accumulation in shoots and grains [16]. These findings underscore the pivotal relationship between the tissue-specific expression of these genes and their physiological functions in roots. Furthermore, the subcellular location of a protein is intimately linked with its function. For instance, both AtNRAMP3 and AtNRAMP4 are localized to the vacuolar membrane [21] and play indispensable roles in maintaining Mn homeostasis [22]. A double mutant of *A. thaliana*, *nramp3nramp4*, accumulates notably greater amounts of Mn in leaf mesophyll cell vacuoles than does the wild type. Notably, OsNRAMP4 (Nrat1) localizes to the plasma membrane of all cells, excluding the epidermal cells of the root tip [23]. This demonstrated the transport of trivalent Al ions in yeast but not other divalent ions, such as Mn^2+^, Fe^2+^, or Cd^2+^ [23]. Knockout of *OsNRAMP4* diminishes rice’s Al intake and intensifies Al binding to cell walls, consequently enhancing Al sensitivity [23]. OsNRAMP5 is located on the root plasma membrane [24] and is actively involved in facilitating the cellular uptake of Cd [25, 26]. In conclusion, the variability in ion selectivity and expression patterns among *NRAMPs* underscores the intricate nature of the physiological functions governed by these genes. Moreover, this complexity is likely compounded by considerations of protein structure and responses to both internal and external stimuli.

Oilseed rape (*Brassica napus*, 2n = 38, AACC) is an allopolyploid species resulting from interspecific hybridization between turnip (*Brassica rapa*, 2n = 20, AA) and Mediterranean cabbage (*Brassica oleracea*, 2n = 18, CC) approximately 7,500 years ago [27]. In contrast to *B. rapa* and *B. oleracea*, *B. napus* has an enlarged NRAMP gene family, suggesting probable diversification in the functional aspects of *NRAMPs* within this particular lineage. However, the functional aspects of *NRAMPs* in Brassica species have not been fully explored. Although *NRAMPs* have been extensively studied in *A. thaliana*, *O. sativa* L., and *M. truncatula*, the existing knowledge concerning their functional roles in these plants falls short of providing a comprehensive understanding of their physiological impacts. To elucidate the intricate functions of *NRAMPs* in Brassica species, further research is warranted, particularly in *B. napus*, considering its pivotal role within the Brassica species. Exploring the evolutionary relationships, functional differentiation, tissue distribution, and responses of gene family members to internal and external cues at the broader family level is crucial for addressing this knowledge gap. Therefore, this study conducted a comprehensive analysis of *NRAMPs* in *B. napus*. Through analysis of publicly available data, 18, 9, and 10 *NRAMPs* were identified in *B. napus*, *B. rapa*, and *B. oleracea*, respectively. This study involved a thorough investigation of the evolutionary relationships, conserved motifs, domains, gene structures, chromosomal positions, cis-regulatory elements, and expression profiles of *NRAMPs* within Brassica species. Furthermore, qRT‒PCR analysis was used to examine the influence of different concentrations of Cd on the expression patterns of the *BnNRAMPs*. Consequently, this research contributes valuable resources toward a thorough comprehension of the evolutionary mechanisms involving *BnNRAMPs*. This study provides valuable insights that may contribute to unraveling the broader physiological functions exhibited by the NRAMP gene family.

## Results

### Identification and evolutionary analysis of the Brassica species NRAMP gene family

Within the genomes of the three Brassica species, a total of 37 *NRAMPs* were identified (Table 1), comprising 18, 9 and 10 *NRAMPs* in *B. napus*, *B. oleracea* and *B. rapa*, respectively. Of the 18 *BnNRAMPs*, 9 were distributed in the A subgenome, while the remaining 9 were distributed in the C subgenome.

**Table 1:** Physicochemical indices and subcellular localization predictions of the *B. napus* NRAMP gene family

No.	Gene ID	Gene name	Chromosome	Protein length (aa^a^)	MW^b^ (kDa^c^)	PI^d^	Subcellular localization predicted
1	BnaA02T0239800ZS	*BnNRAMP1.1*	A02	532	57671.20	8.69	Plas^e^
2	BnaA03T0240800ZS	*BnNRAMP3.1*	A03	511	56462.12	5.11	Plas
3	BnaA03T0450100ZS	*BnNRAMP5.1*	A03	532	58569.24	5.00	Plas
4	BnaA06T0107800ZS	*BnNRAMP6.1*	A06	498	53854.77	9.17	Plas
5	BnaA07T0140800ZS	*BnNRAMP4.1*	A07	512	56172.97	5.25	Plas
6	BnaA07T0228600ZS	*BnNRAMP1.2*	A07	527	57213.62	8.83	Plas
7	BnaA07T0385500ZS	*BnNRAMP1.3*	A07	519	55885.99	7.98	Plas
8	BnaA08T0046600ZS	*BnNRAMP2.1*	A08	527	57949.84	5.26	Plas
9	BnaA10T0059400ZS	*BnNRAMP2.2*	A10	532	58343.27	5.20	Plas
10	BnaC02T0323200ZS	*BnNRAMP1.4*	C02	532	57692.27	8.79	Plas
11	BnaC03T0284400ZS	*BnNRAMP3.2*	C02	511	56487.12	5.11	Plas
12	BnaC05T0133400ZS	*BnNRAMP6.2*	C05	494	53253.95	9.06	Plas
13	BnaC06T0018700ZS	*BnNRAMP2.3*	C06	533	58551.53	5.21	Plas
14	BnaC06T0246600ZS	*BnNRAMP1.5*	C06	543	59046.65	8.78	Plas
15	BnaC06T0453300ZS	*BnNRAMP1.6*	C06	519	55922.17	7.58	Plas
16	BnaC07T0204600ZS	*BnNRAMP4.2*	C07	511	56119.95	5.25	Plas
17	BnaC07T0425700ZS	*BnNRAMP5.2*	C07	531	58567.37	5.18	Plas
18	BnaC08T0059700ZS	*BnNRAMP2.4*	C08	532	58590.57	5.18	Plas
19	BolC02g035780.2 J.m1	*BoNRAMP1.1*	C02	532	57653.18	8.69	Plas
20	BolC03g029960.2 J.m1	*BoNRAMP3.1*	C03	511	56487.12	5.11	Plas
21	BolC05g013610.2 J.m1	*BoNRAMP6*	C05	504	54517.42	8.98	Plas
22	BolC06g001670.2 J.m1	*BoNRAMP2.1*	C06	535	58787.75	5.16	Plas
23	BolC06g028220.2 J.m1	*BoNRAMP1.2*	C06	529	58152.75	9.08	Plas
24	BolC06g050660.2 J.m1	*BoNRAMP1.3*	C06	546	59054.01	7.97	Plas
25	BolC07g023330.2 J.m1	*BoNRAMP4*	C07	511	56119.95	5.25	Plas
26	BolC07g048030.2 J.m1	*BoNRAMP5*	C07	531	58621.50	5.18	Plas
27	BolC08g006720.2 J.m1	*BoNRAMP2.2*	C08	527	57995.87	5.18	Plas
28	BolC08g047980.2 J.m1	*BoNRAMP3.2*	C08	519	57021.42	4.84	Plas
29	BraA02g026430.3.5 C.1	*BrNRAMP1.1*	A02	532	57671.20	8.69	Plas
30	BraA03g025990.3.5 C.1	*BrNRAMP3*	A03	518	57251.87	5.16	Plas
31	BraA03g049130.3.5 C.1	*BrNRAMP5*	A03	532	58580.27	5.00	Plas
32	BraA06g012000.3.5 C.1	*BrNRAMP6*	A06	452	48937.31	9.16	Plas
33	BraA07g017080.3.5 C.1	*BrNRAMP4*	A07	512	56172.97	5.25	Plas
34	BraA07g026950.3.5 C.1	*BrNRAMP1.2*	A07	347	37788.77	8.87	Vacu^f^
35	BraA07g043660.3.5 C.1	*BrNRAMP1.3*	A07	571	61946.47	7.92	Plas
36	BraA08g005430.3.5 C.1	*BrNRAMP2.1*	A08	527	57975.82	5.18	Plas
37	BraA10g006820.3.5 C.1	*BrNRAMP2.2*	A10	532	58343.27	5.20	Plas

*Notes* aa^a^: Amino acid; MW^b^: Molecular weight; kDa^c^: KiloDalton; PI^d^: Isoelectric point; Plas^e^: Plasma membrane; Vacu^f^: Vacuole

Physicochemical property analysis of NRAMP proteins is valuable for predicting their structure, function, protein interactions, and evolutionary relationships. Among the 37 identified NRAMP proteins in Brassica species, all of the proteins exhibited hydrophobic properties, reflecting their role as transporters. The majority of the NRAMP proteins were stable (with an instability coefficient < 40), except for five unstable proteins (13.51%). Subfamily I (NRAMP1s/6s) had smaller average sequence lengths and molecular weights (511 aa and 55.39 kD, respectively) compared to the larger sequence lengths and molecular weights (523 aa and 57.58 kD, respectively) observed in the subfamily II (NRAMP2s/3s/4s/5s). The remaining subfamily I proteins were alkaline (average theoretical isoelectric point of 8.64), and those in subfamily II were acidic (average theoretical isoelectric point of 5.61). Despite substantial differences in isoelectric points between the two subgroups, the majority of the NRAMP protein regions were hydrophobic, suggesting minimal differences in the actual charge properties of the proteins.

Protein localization within the cell is intricately linked to protein function; therefore, predicting the cellular localization of a protein is indispensable for investigating gene function. Subcellular localization prediction using WoLFPSORT indicated that all NRAMP proteins in the three Brassica species were localized on the plasma membrane, except for BrNRAMP1.2, which was located in vacuoles. This finding suggested that the primary function of Brassica species NRAMP proteins may involve regulating ion homeostasis inside and outside the cell. However, predictions from the Cell-PLoc 2.0 tool placed BrNRAMP1.2 on the plasma membrane, indicating that more accurate subcellular localization requires further experimental validation. Overall, the predicted subcellular location consolidates the transporter activity of NRAMPs, yet BrNRAMP1.2 may perform unique functions in metal ion homeostasis.

**Fig. 1:**
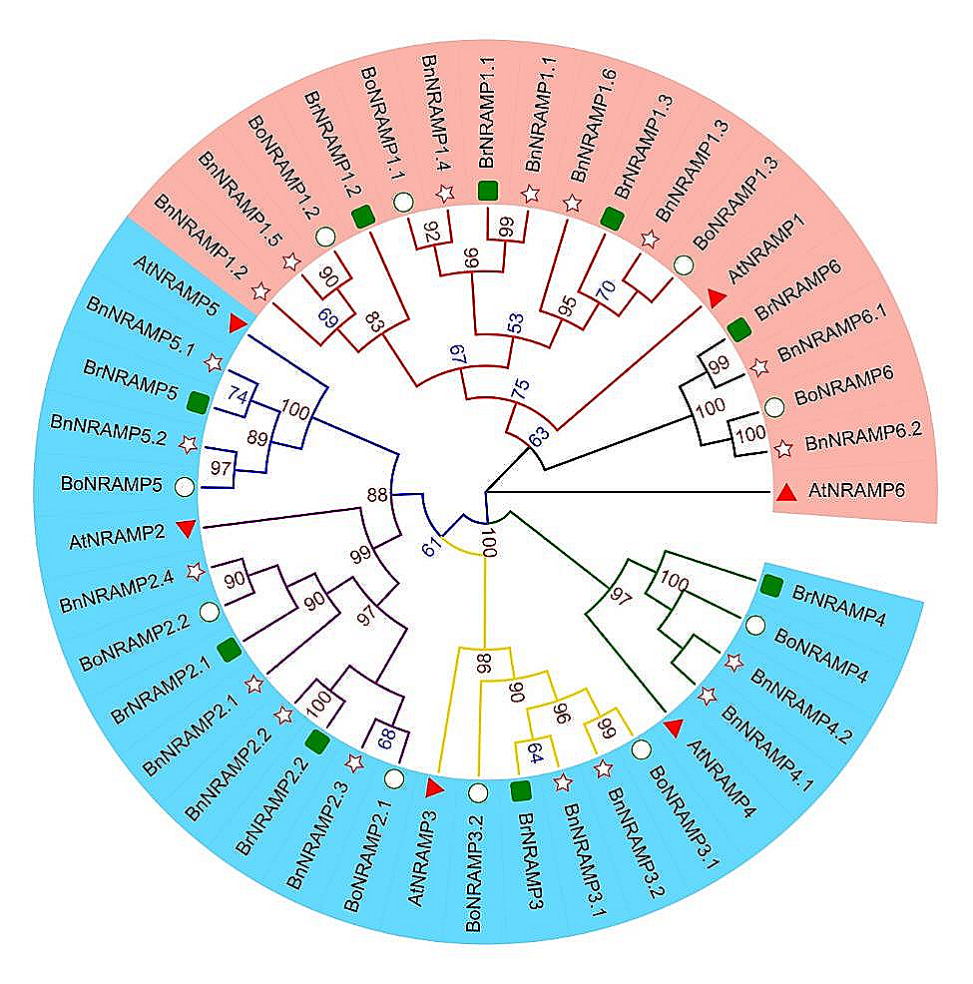
Phylogenetic tree of *B. napus*, *B. rapa*, *B. oleracea*, and *A. thaliana*. In this diagram, the light orange leaf background delineates subfamily I (NRAMP1s/6s), while the deep sky-blue leaf background signifies subfamily II (NRAMP2s/3s/4s/5s). Branches are color-coded for clarity: NRAMP6s are represented by black, NRAMP1s by red, NRAMP5s by blue, NRAMP2s by purple, NRAMP3s by gold, and NRAMP4s by green. With respect to leaf label decoration, different shapes indicate distinct NRAMPs of various plant species: triangles denote AtNRAMPs, stars denote BnNRAMPs, circles denote BoNRAMPs, and rectangles denote BrNRAMPs. Moreover, it is pertinent to emphasize that bootstrap values ranging from 80 to 100 are distinctly marked in a dark red shade, those between 50 and 80 are indicated in blue, while bootstrap values falling within the range of 0 to 50 are not rendered for display

Evolutionary analysis is highly beneficial for studying gene functions, interspecies evolutionary relationships, genetic diversity and variations. To determine the evolutionary relationships between the *A. thaliana*, *B. napus*, *B. rapa*, and *B. oleracea* NRAMP gene families, we constructed a phylogenetic tree. The results revealed that all the Brassica species *NRAMPs* clustered well with their homologous genes in *A. thaliana* (Fig. 1). Based on phylogenetic relationships, the NRAMP genes in Brassica species were categorized into two subfamilies: NRAMP1s/6s constituted subfamily I, whereas NRAMP2s/3s/4s/5s comprised subfamily II.

In the genomes of *B. rapa*, *B. oleracea*, and *B. napus*, the homologous genes to *AtNRAMP1* were found as 3 (*BrNRAMP1.1/1.2/1.3*), 3 (*BoNRAMP1.1/1.2/1.3*), and 6 (*BnNRAMP1.1/1.2/1.3/1.4/1.5/1.6*), respectively. Similarly, homologous genes to *AtNRAMP2* were observed as 2 (*BrNRAMP2.1/2.2*), 2 (*BoNRAMP2.1/2.2*), and 4 (*BnNRAMP1.1/1.2/1.3/1.4*), while genes homologous to *AtNRAMP4*/*5*/*6* were detected as 1 (*BrNRAMP4; BrNRAMP5; BrNRAMP6*), 1 (*BoNRAMP4; BoNRAMP5; BoNRAMP6*), and 2 (*BnNRAMP4.1/4.2; BnNRAMP5.1/5.2; BnNRAMP6.1/6.2*) ,respectively, across these genomes. The number of genes homologous to *AtNRAMP1/2/4/5/6* in the *B. napus* genome equals the sum of such homologous genes found in the genomes of *B. rapa* and *B. oleracea*. Moreover, the analysis revealed the presence of 1 (*BrNRAMP3*), 2 (*BoNRAMP3.1, BoNRAMP3.2*), and 2 (*BnNRAMP3.2, BnNRAMP3.2*) orthologous genes to *AtNRAMP3* in the *B. rapa*, *B. oleracea*, and *B. napus* genomes, respectively. The phylogenetic analysis depicted in Fig. 1 reveals the presence of two distinct *BnNRAMP3* genes, one originating from *BrNRAMP3* and the other from *BoNRAMP3.1*. Notably, *BoNRAMP3.2* in *B. oleracea* has no orthologous genes in *B. napus*, suggesting that this gene was lost during the evolution of *B. napus*.

### Chromosomal localization and collinearity analysis of the Brassica species NRAMP gene family

**Fig. 2:**
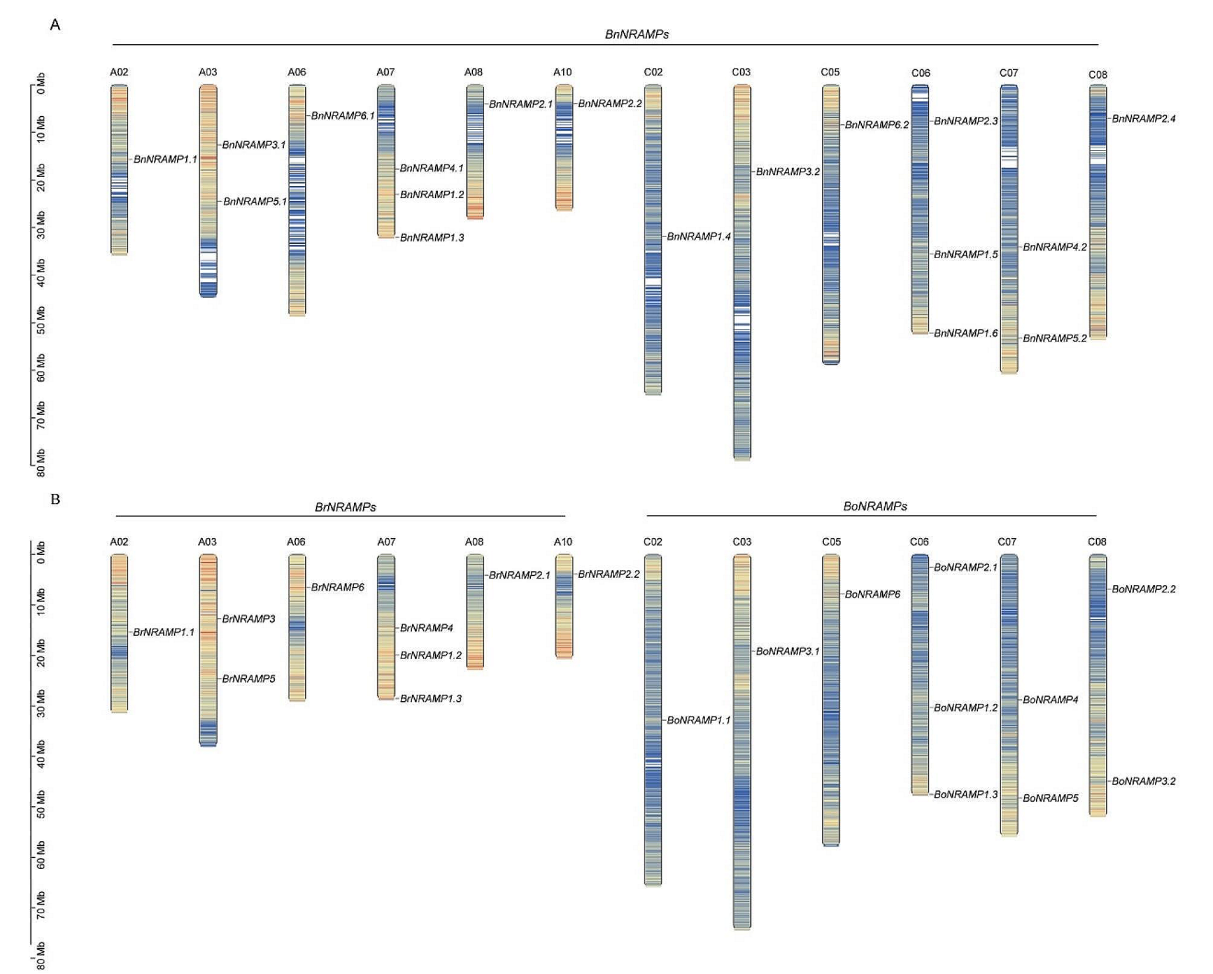
Chromosomal localization of the Brassica species NRAMP gene family. (**A**) Chromosomal localization in *B. napus*. (**B**) Chromosomal localization in *B. rapa* and *B. oleracea*

Chromosomal localization revealed that 18 *BnNRAMPs* were distributed across 12 out of the 19 chromosomes in the *B. napus* genome, with 9 in each A and C subgenome (Fig. 2A, Additional file 1). In *B. rapa*, 9 *BrNRAMPs* are located on 6 chromosomes out of 10, and in *B. oleracea*, 10 *BoNRAMPs* are positioned on 7 chromosomes out of 9 (Fig. 2B, Additional file 1). The number of *NRAMP* genes on each chromosome ranged from 1 to 3, indicating that there was no apparent correlation with chromosome length. The dispersed arrangement of *NRAMP* genes on chromosomes suggested that these genes did not form gene clusters.

**Fig. 3:**
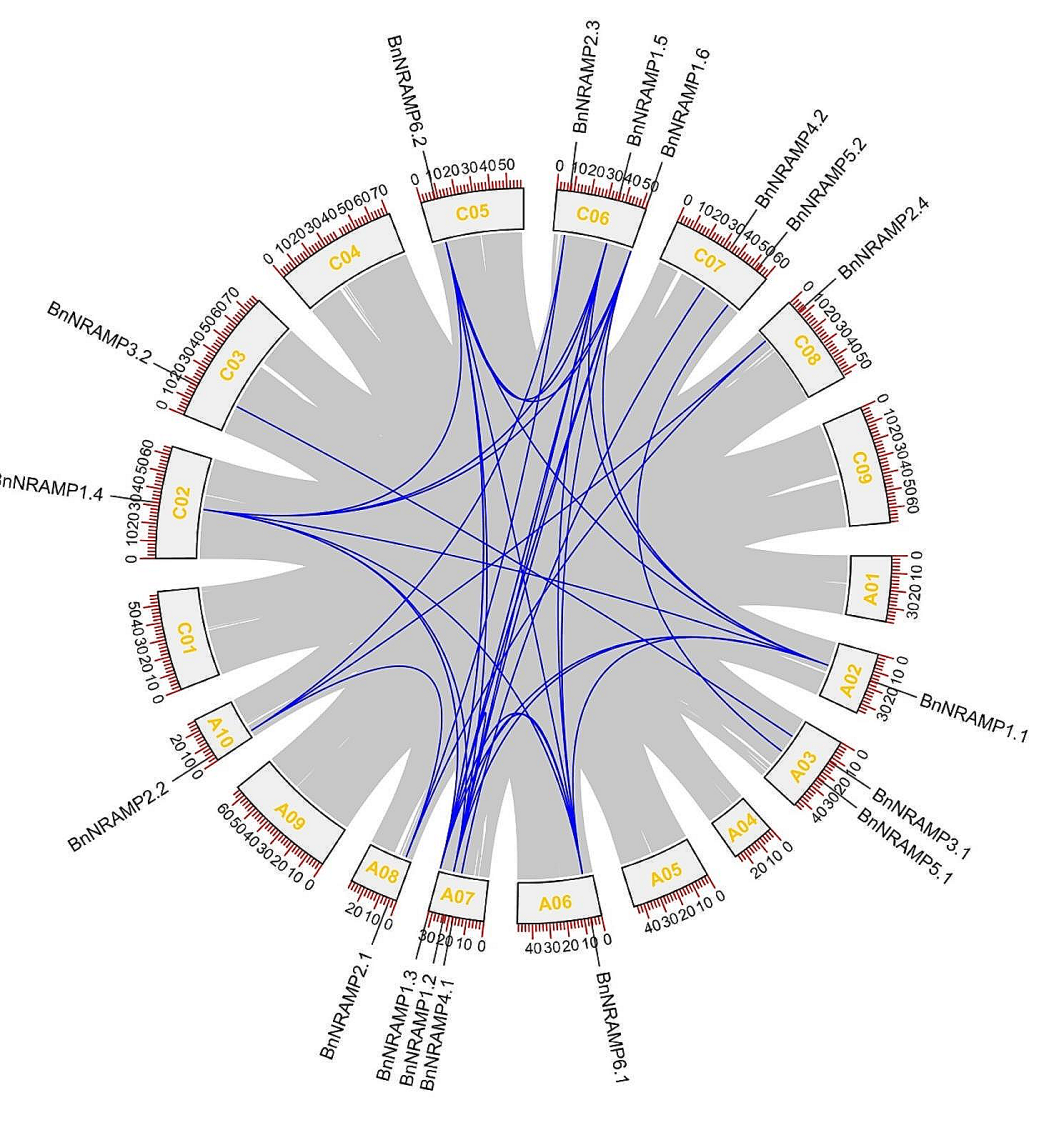
Collinearity relationships within the *B. napus* genome. The gray lines in the background indicate colinear blocks among Brassica species, while the blue lines represent collinear *NRAMP* gene pairs

Collinearity analysis serves as a pivotal tool for comprehensively exploring genome architecture and evolution, facilitating the elucidation of genetic relationships and evolutionary trajectories among diverse biological species. An examination of collinearity within *B. napus* revealed 37 *NRAMP* syntenic gene pairs (Fig. 3).

**Fig. 4:**
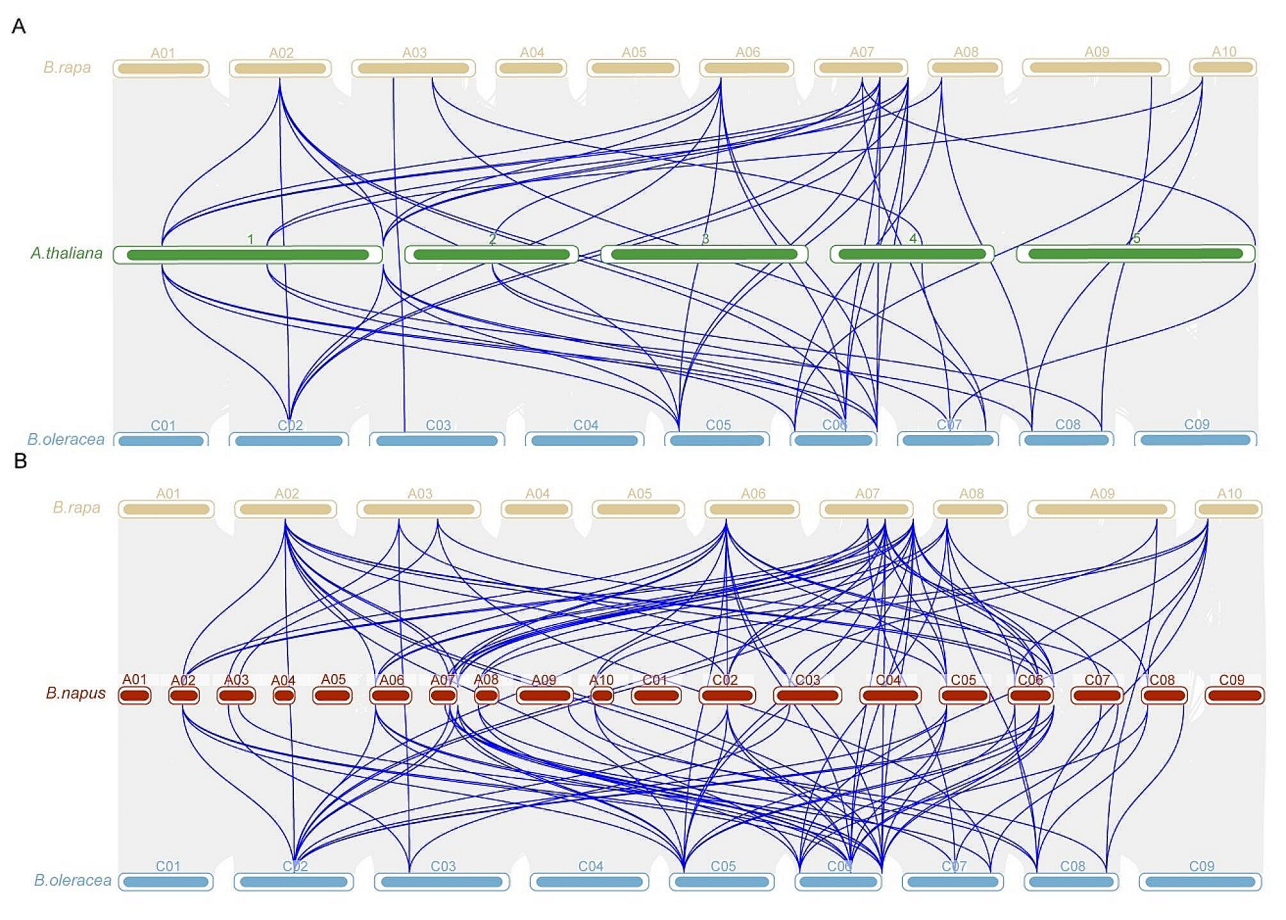
Collinearity relationships of *NRAMPs* among *B. napus* and three ancestral plants. (**A**) Relationships of collinearity among the *A. thaliana*, *B. rapa*, and *B. napus* genomes. (**B**) Collinearity relationships among the *B. rapa*, *B. napus*, and *B. oleracea* genomes. The gray lines in the background indicate colinear blocks among Brassica species, while the blue lines represent collinear *NRAMP* gene pairs

Furthermore, intergenomic collinearity analysis involving *A. thaliana*, *B. rapa*, and *B. oleracea* revealed 50 *NRAMP* syntenic gene pairs (Fig. 4A), while 115 *NRAMP* syntenic gene pairs were identified in the collinearity analysis among *B. napus*, *B. rapa*, and *B. oleracea* (Fig. 4B).

In plant genomes, tandem repeats and segmental duplications have been instrumental in expanding gene family members and facilitating the emergence of novel functions during evolutionary processes [28]. To elucidate the evolutionary scenarios within the NRAMP gene families of *B. napus*, *B. rapa*, and *B. oleracea*, we investigated tandem repeats and segmental duplication events. Surprisingly, no tandem repeat genes were observed in *B. napus*, *B. rapa*, or *B. oleracea*. Among the 37 Brassica species *NRAMP* genes studied, all were found to have originated from whole-genome duplication or segmental duplication events (Additional file 2). These findings strongly indicate the pivotal role of segmental duplication in the evolutionary trajectory of *NRAMP* genes.

The evaluation of positive selection pressure on recurrent events relies on nonsynonymous (Ka) and synonymous (Ks) substitution rates. This study computed the Ka/Ks ratios between the *NRAMP* genes in *B. napus* and those in *B. rapa* and *B. oleracea*. The Ka/Ks values ranged from 0.15 to 0.60, with an average of 0.31. Notably, all the *NRAMP* genes exhibited Ka/Ks values less than 1 (Additional file 3), suggesting that the evolution of *NRAMP* genes in *B. napus* occurred under the influence of purifying selection.

### Conserved motifs, domains, and gene structure analysis of the Brassica species NRAMP gene family

**Fig. 5:**
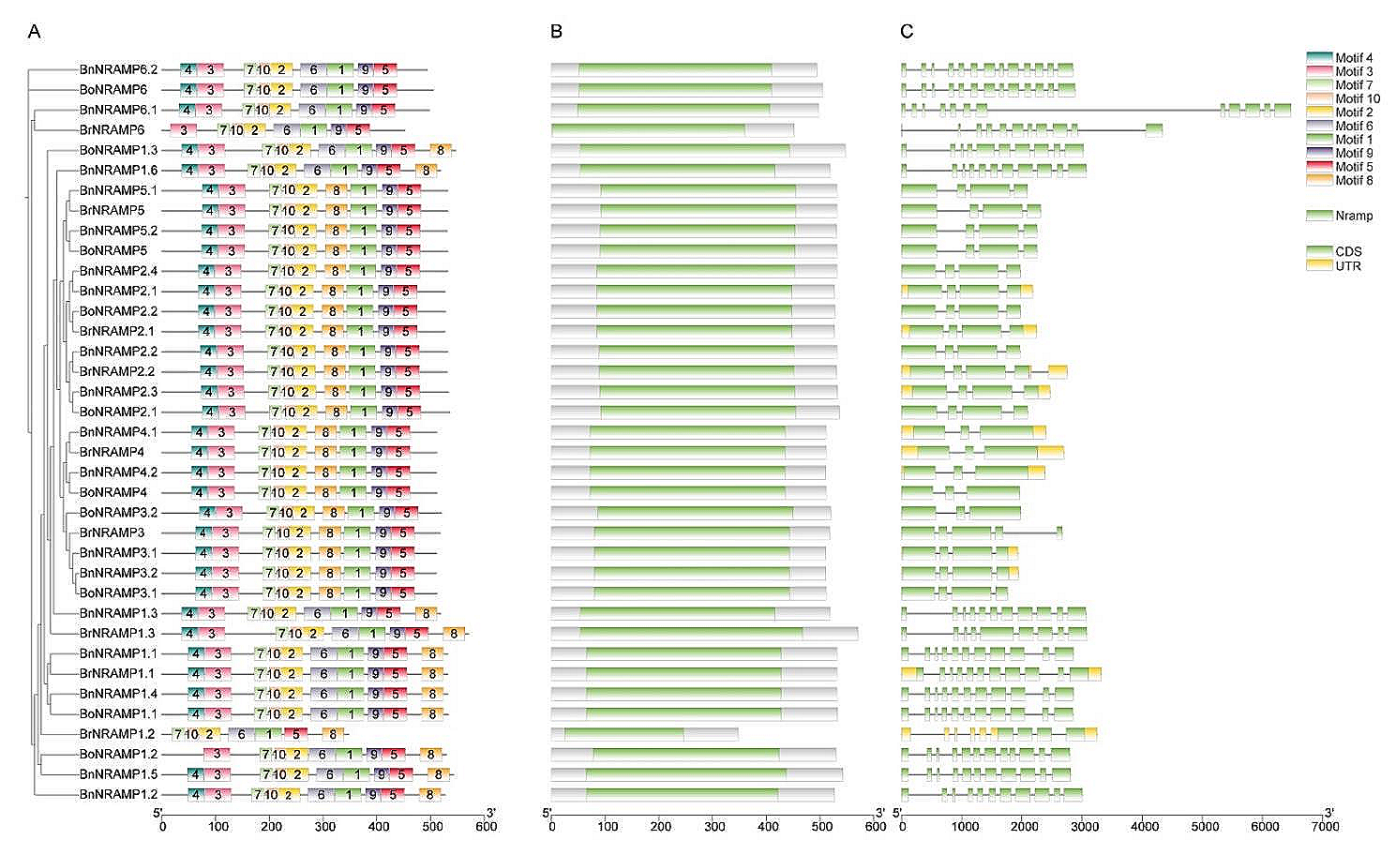
Conserved motif, domain, and gene structure analysis of *B. napus*, *B. rapa*, and *B. oleracea* NRAMP genes. (**A**) Conserved motif analysis of protein sequences. (**B**) Domain analysis. (**C**) Gene structure analysis

To predict protein function and discover the relationship between protein structure and function, conserved motif analysis was performed. An examination of the conservation patterns within the protein sequences of the Brassica species NRAMP gene family revealed several conserved motifs, and the distributions of the top 10 highly conserved motifs are shown in Fig. 5A. All NRAMP proteins contained motifs 1, 2, 5, and 7 within the central region of their protein sequences, indicating a high level of conservation of these motifs within the NRAMP gene family of Brassica species. This finding underscores the importance of these motifs for the NRAMP gene family. The number, type, and distribution of motifs within the NRAMP gene family in Brassica species exhibit considerable variation. With the exception of BrNRAMP1.2 (7 motifs), BoNRAMP1.2 (9 motifs), and BrNRAMP6 (8 motifs), the remaining NRAMP proteins feature 10 motifs. This finding underscores the unique functional characteristics of these NRAMPs compared to those of other NRAMPs. Among the NRAMP family proteins, all members of subfamily I (NRAMP1/6) harbor motif 6, while members of subfamily II (NRAMP2/3/4/5) lack this motif. Furthermore, except for NRAMP6s, all the other NRAMPs contained motif 8. Notably, both NRAMP1s and NRAMP2s/3s/4s/5s harbor motif 8, yet the positioning of motif 8 varies between these two subclasses of NRAMPs. In NRAMP1s, motif 8 spans amino acids 450–600, whereas in NRAMP2s/3s/4s/5s, motif 8 is located between amino acids 250–350. Hence, Motif 6 and motif 8 could be pivotal contributors to the functional distinctions observed between subfamilies I and II within the NRAMP gene family. Conducted conserved domain analysis of the 37 Brassica species NRAMP protein sequences using the Pfam and NCBI databases revealed that all the NRAMP proteins possess a conserved domain labelled “Nramp,” representing the hallmark domain of the NRAMP gene family (Fig. 5B). Additionally, except for motif 8 of NRAMP1s, all the other motifs reside within this conserved domain.

A comparison of gene structures within the Brassica species NRAMP gene family (Fig. 5C) revealed significant variations in gene length and the number of introns. Genes with high sequence similarity exhibit a similar number of exons, as well as similar lengths of exons and introns. The maximum sequence length among the Brassica species NRAMP gene family sequences was 6474 bp (*BnNRAMP6.1*), while the minimum was 1762 bp (*BoNRAMP3.1*). The number of exons in the Brassica species NRAMP gene family ranged from 3 to 13. Intriguingly, the sequences of subfamily I genes (averaging 3285 bp) are longer than those of subfamily II genes (averaging 2208 bp). Similarly, the number of exons in subfamily I genes (averaging 11) exceeded that in subfamily II genes (averaging 4). Interestingly, the coding sequence (CDS) lengths of either subfamily I or subfamily II are not distinguishable from each other, with approximately 30 nt more in clade I. The larger exon numbers in subfamily I are likely counteracted by the smaller average size of exons, indicating elasticity in alternative splicing. This scenario results in enrichment of transcripts and proteins, suggesting a dynamic regulatory mechanism at the posttranscriptional level.

### Cis-acting element analysis of the Brassica species NRAMP gene family promoter regions

**Fig. 6:**
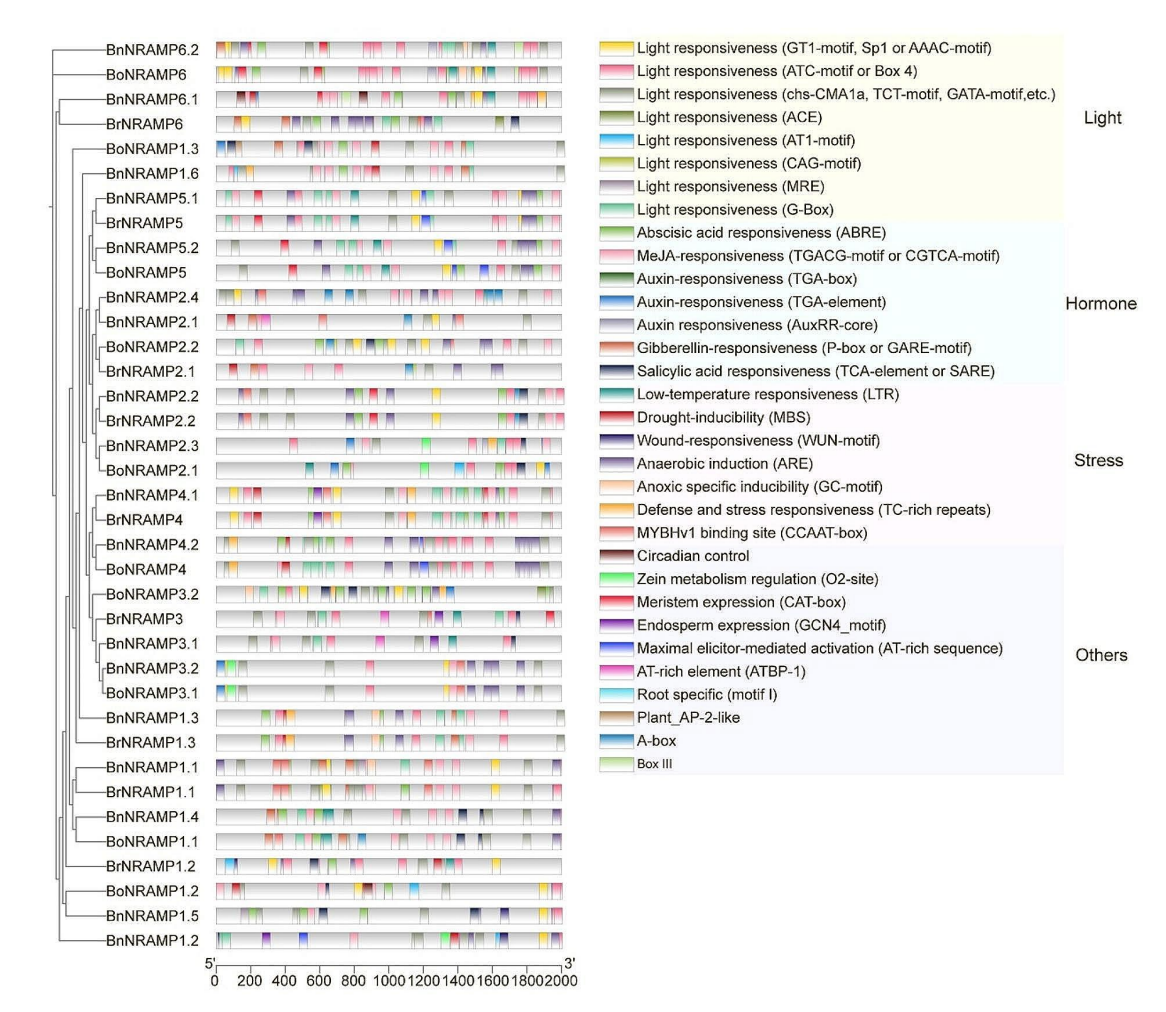
Cis-regulatory element analysis of the selected 2000 bp upstream promoter regions of the Brassica species *NRAMP* genes

Understanding cis-regulatory elements is pivotal in predicting gene functions and unravelling the intricate mechanisms governing gene expression regulation. The NRAMP gene family is significantly impacted by diverse abiotic stresses. To assess the potential functions of *NRAMP* genes within Brassica species, a comprehensive analysis and screening of cis-regulatory elements in the promoter regions—comprising the 2000 upstream base pairs of *NRAMP* genes—were conducted. Our investigation revealed several cis-regulatory elements intricately associated with regulating abiotic stresses and hormonal responses, as depicted in Fig. 6. Notably, substantial variability exists within the NRAMP gene family concerning both the quantity and types of cis-regulatory elements. In terms of quantity, *BoNRAMP3.2* exhibited the highest abundance of cis-regulatory elements (48 elements), while *BnNRAMP2.1* had the lowest (12 elements). In terms of diversity, *BnNRAMP1.2* demonstrated the most expansive repertoire (15 elements), whereas *BrNRAMP2.1* showed the most limited set (8 elements). Empirical studies suggest a potential correlation between abscisic acid (ABA) and a reduction in Cd uptake via the downregulation of *NRAMP* expression [5]. Our detailed analysis revealed that, with the exception of 7 genes—*BnNRAMP1.6/2.1/2.4/3.2*, *BoNRAMP1.3/3.1*, and *BrNRAMP2.1*—the promoters of most Brassica species *NRAMP* genes (30 elements for ABA, 32 elements for MeJA) contain response elements for ABA and methyl jasmonate (MeJA). Furthermore, a subset of Brassica species *NRAMP* gene promoters lacking ABA response elements shows responsiveness to other plant hormones, such as gibberellin (GA), salicylic acid (SA), and auxin. This finding suggested the potential induction of Brassica species *NRAMP* genes by hormones, particularly ABA and MeJA. Considering the significant correlation of ABA and MeJA with abiotic and biotic stress [3], these compounds may influence NRAMP family gene expression, potentially impacting metal ion absorption under adverse environmental conditions. Moreover, the *NRAMP* gene promoters of 37 Brassica species contain light-responsive elements, indicating the potential of light to modulate *NRAMP* gene expression due to its role in maintaining element homeostasis, which is crucial for photosynthesis-related elements such as Fe, Mn, magnesium (Mg), calcium (Ca), and copper (Cu). Additionally, aerobic respiration in plant cells, which is crucial for ion uptake, requires adequate energy supplied by aerobic respiration. The promoter regions of most Brassica species *NRAMP* genes (31 genes) contain anaerobic responsive elements (AREs), further supporting their association with plant tolerance to abiotic stress, particularly anaerobic conditions. Importantly, 15 *NRAMP* gene promoters harboured defence and stress response elements, 26 harboured MYB transcription factor response elements, 17 had SA response elements, 16 had cold response elements, and 14 had drought-induced response elements. These findings underscore the diverse and potential multifaceted roles of the NRAMP gene family in various biological and abiotic stress responses.

### Analysis of the expression patterns of *BnNRAMPs* based on transcriptome data

**Fig. 7:**
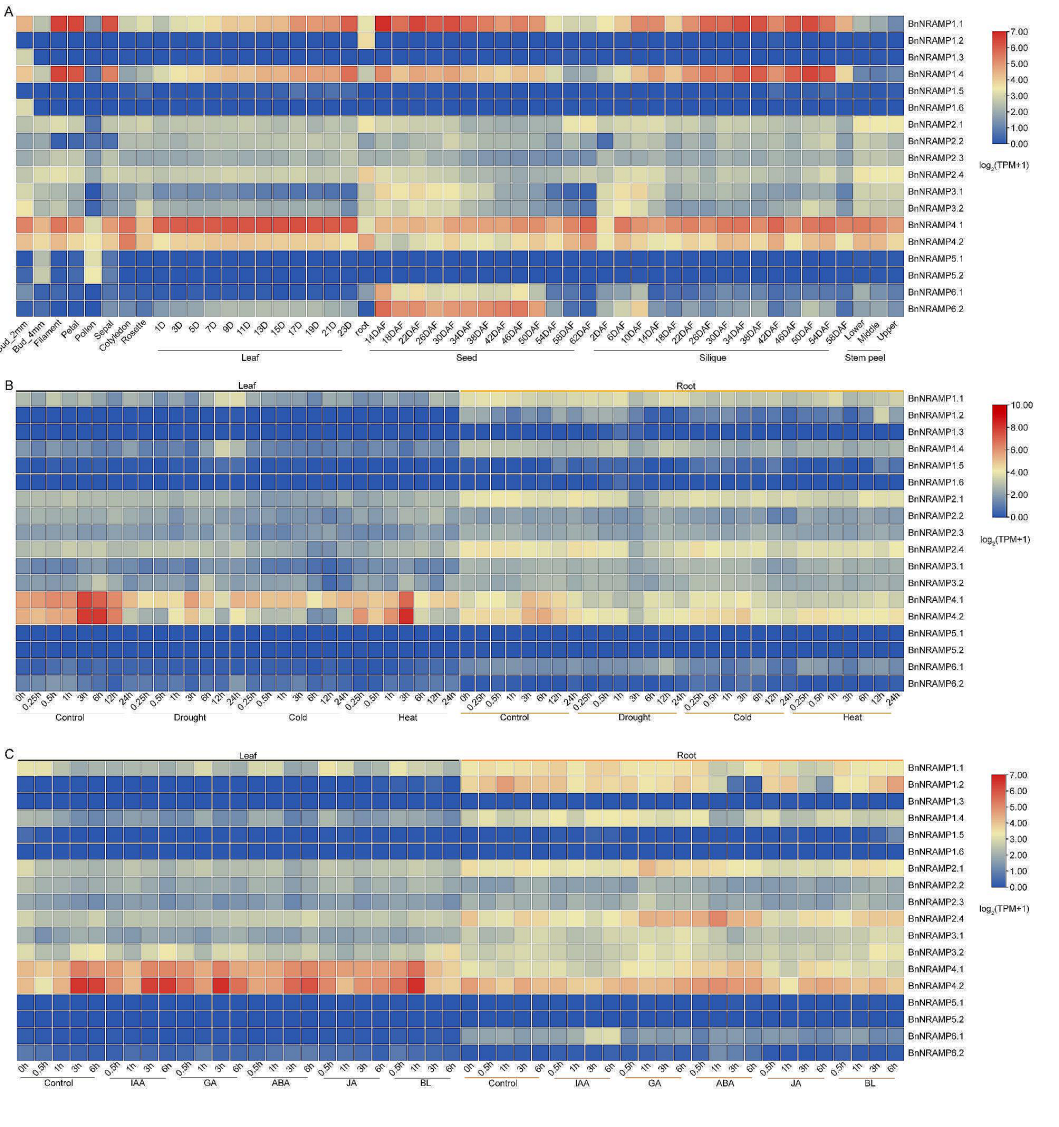
Analysis of expression patterns in *BnNRAMPs*. (**A**) Tissue-specific expression patterns of *BnNRAMPs*. (**B**) Expression patterns of *BnNRAMPs* under stress conditions. (**C**) Expression patterns of *BnNRAMPs* under hormone treatments. In Fig. 7A, the notations ‘N + D’ and ‘N + DAF’ represent the Nth day and the Nth day after flowering, respectively. Within Fig. 7C, the abbreviations correspond to specific phytohormones: IAA, indole-3-acetic acid; GA, gibberellic acid; ABA, abscisic acid; JA, jasmonic acid; and BL, brassinolide. The expression of the *BnNRAMPs* was normalized and represented as transcripts per kilobase of exon model per million mapped reads (TPM) values, and the log_2_(TPM + 1) was used to construct the heatmap diagram

The expression patterns of genes represent a pivotal aspect of elucidating gene function. To explore the roles of the *NRAMP* genes further, we constructed a heatmap displaying *NRAMP* gene expression patterns utilizing publicly available data from the BnIR website (https://yanglab.hzau.edu.cn/). Notably, *BnNRAMP1.2* exhibited minimal expression in roots, whereas *BnNRAMP1.3/1.6* exhibited slight expression in 2 mm buds (Fig. 7A). In contrast, *BnNRAMP5.1/5.2* demonstrated minimal expression in 4 mm buds and pollen. Intriguingly, *BnNRAMP1.1* exhibited elevated expression in stamens, petals, sepals, seeds (at 14–34 days postflowering), and siliques (at 26–54 days postflowering). Similar expression patterns were observed for *BnNRAMP1.4*. Moreover, *BnNRAMP4.1* exhibited expression across multiple tissues, including buds, petals, leaves, roots, seeds, and siliques, with notably greater expression in leaves. Concurrently, *BnNRAMP6.2* exhibited increased expression in seeds between 14 and 50 days after flowering, followed by nearly undetectable expression levels from 54 to 62 days after flowering. This finding delineates the functional involvement of this gene across diverse tissues and developmental stages, underscoring the significance of ion uptake throughout the life cycle of *B. napus*. Expression analyses revealed that *BnNRAMPs*, particularly *BnNRAMP1.2/1.3/1.5/1.6/5.1/5.2/6.1/6.2*, were scarcely detected under drought, cold, or heat stress (Fig. 7B). Conversely, the expression of *BnNRAMP4.1/4.2* in leaves and roots decreased during exposure to these adverse conditions. Overall, the *BnNRAMP* genes function in coordination to withstand diverse environmental conditions. The assessment of *B. napus NRAMP* gene responses to various hormone treatments revealed a lack of notable sensitivity across different types of hormones (Fig. 7C). Notably, under hormonal treatment, *BnNRAMP1.3/1.5/1.6/5.1/5.2/6.2* exhibited low and unresponsive expression levels. Conversely, *BnNRAMP4.1/4.2* displayed increased expression within 0.5–1 h after treatment with indole-3-acetic acid (IAA), GA, ABA, and jasmonic acid (JA). However, their expression levels decreased, reaching or falling below those of the control group at 3–6 h posttreatment. Interestingly, unlike those of other hormones, the expression of *BnNRAMP4.1/4.2* substantially increased within 0.5–1 h following treatment with brassinolide (BL), followed by a pronounced decrease at 3–6 h. This finding underscores the heightened sensitivity of *BnNRAMPs*, particularly *BnNRAMP4.1/4.2*, to BL compared to that of other hormones.

### Analysis of the expression patterns of *BnNRAMPs* under cadmium stress

**Fig. 8:**
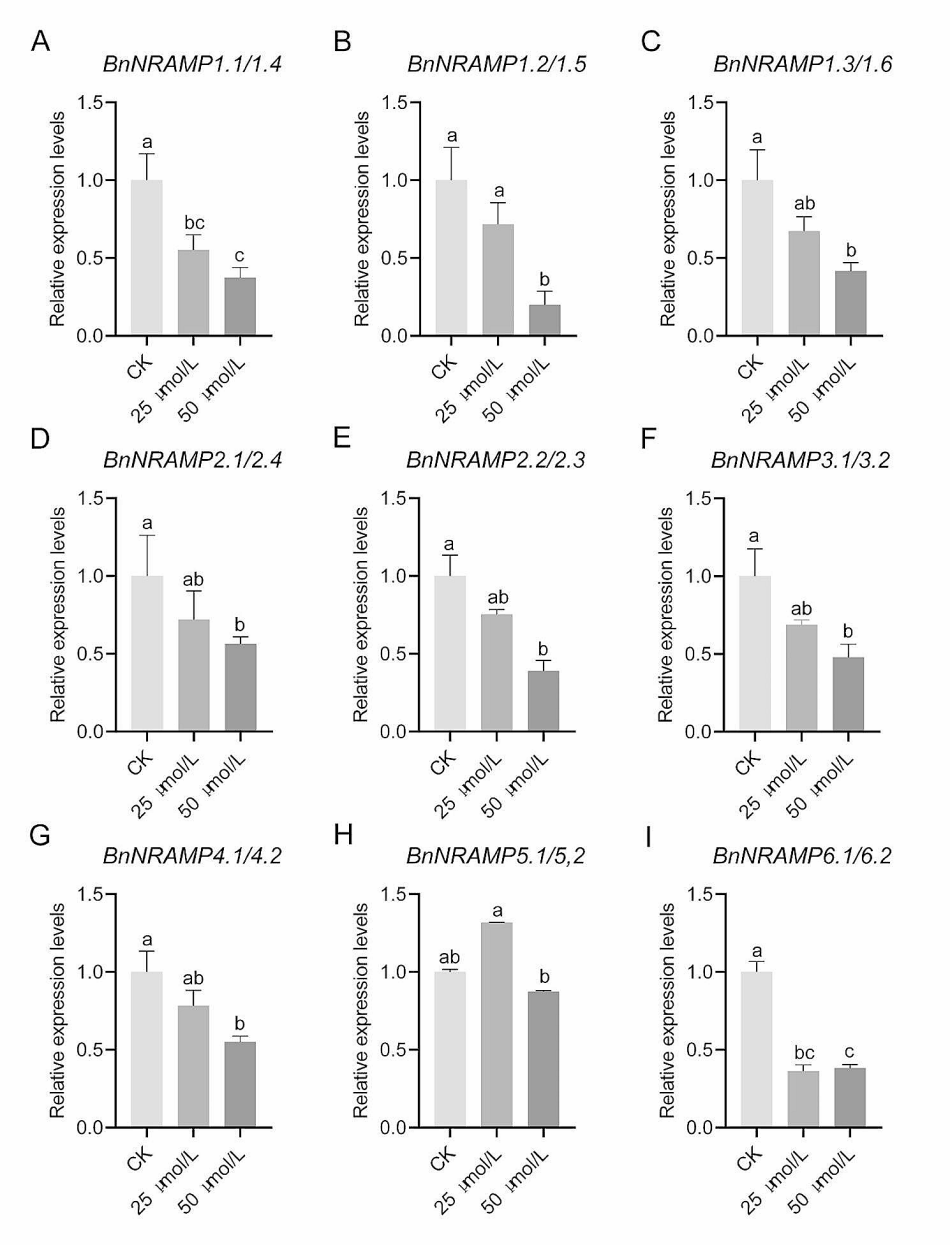
Differential responses of *BnNRAMP* expression under cadmium stress in *B. napus* seedlings. Figure 8 (**A**-**I**) shows the *BnNRAMPs*. The expression level of each gene in the control plants at 0 µmol/L was normalized to 1.0. Error bars represent the mean values of three replicates ± SEM (standard error of the mean). Different lowercase letters indicate significant differences according to Duncan’s multiple range test (*p* < 0.05)

In this study, the impact of varying concentrations of CdCl_2_·5/2H_2_O (25 µmol/L and 50 µmol/L) on the expression of *BnNRAMPs* in *B. napus* seedlings was investigated. Our findings revealed distinct expression patterns among *BnNRAMPs* under Cd stress conditions (Fig. 8). Most *BnNRAMPs* exhibited decreased expression levels in response to Cd treatment compared to those in the control group. The observed decrease in expression levels with increasing Cd concentrations suggested a dose-dependent effect, presumably influenced by cadmium-induced toxicity impacting the regulatory mechanisms of these transporters. Similarly, *BnNRAMP6.1/6.2* presented reduced expression levels under Cd treatment, consistent with the overall trend. Nevertheless, a marginal upregulation in expression was noted for *BnNRAMP6.1/6.2* at relatively high Cd concentrations (50 µmol/L), suggesting a nuanced response under elevated stress conditions. However, compared to the low Cd treatment, the expression levels of *BnNRAMP6.1/6.2* were relatively higher under the 50 µmol/L Cd treatment, indicating a potentially finer regulation in *B. napus* under high cadmium concentrations. Notably, *BnNRAMP5.1/5.2* exhibited a divergent response mechanism compared to that of other *BnNRAMPs* under Cd stress. In contrast to the general trend, *BnNRAMP5.1/5.2* exhibited increased expression in response to 25 µmol/L Cd, implying heightened sensitivity to lower Cd levels and a potential role in responding to reduced ionic strength. Nevertheless, after 50 µmol/L Cd treatment, phytotoxicity was evident in *B. napus* seedlings, leading to a subsequent decrease in the expression of *BnNRAMP5.1/5.2*, highlighting the intricate interplay between Cd stress levels and gene expression regulation. These observations shed light on the intricate regulatory mechanisms governing *BnNRAMP* expression under Cd stress, emphasizing the need for further exploration into the specific molecular responses of these transporters in *B. napus* under varying stress conditions.

## Discussion

Elucidation of the mechanisms governing Cd absorption and transport is imperative. Although the association between NRAMP transporters and Cd absorption has been documented in certain species, such as *A. thaliana* and *O. sativa* L [14, 17, 29]. , advancements in this area of research remain notably limited. Additionally, investigations elucidating the involvement of NRAMP transporters in the uptake and translocation of Cd in *B. napus* are scarce. Hence, this study undertook a comprehensive analysis of the NRAMP gene family within Brassica species using bioinformatics methodologies. Our objective was to provide novel insights into the function of the NRAMP gene family within Brassica species.

In this investigation, a total of 37 *NRAMP* genes were discerned within the Brassica species genome and distributed across its varieties, such as *Brassica napus* (18 genes), *B. rapa* (9 genes), and *B. oleracea* (10 genes). Additionally, in other previously documented species, namely, *A. thaliana* [30], *O. sativa* L [31]. , *Glycine max* [32], and *Arachis hypogaea* [33], The presence and number of *NRAMP* genes were noted for 6 genes, 7 genes, 13 genes, and 15 genes. These results underscore the conserved origin of the NRAMP gene family across diverse plant species. However, the number of genes substantially varies among the abovementioned species. Therefore, despite their common ancestry, it is likely that environmental stresses were distinctly exerted on these species, resulting in different evolutionary pathways of the *NRAMP* family, which ultimately led to different scales of gene numbers. Based on phylogenetic analysis, the 37 Brassica species *NRAMP* genes were categorized into two distinct subfamilies: subfamily I, comprising *NRAMP1s* and *NRAMP6s*; and subfamily II, encompassing *NRAMP2s*, *NRAMP3s*, *NRAMP4s*, and *NRAMP5s*. This classification aligns with the established categorization of the NRAMP gene family observed in *A. thaliana* [30]. Owing to the whole-genome triplication of Brassica species [34, 35], the *A. thaliana* gene typically corresponds to three homologues in *B. rapa* or *B. oleracea*. However, except for *NRAMP1s*, the numbers of other *NRAMPs* in *B. rapa* and *B. oleracea* is less than three times. Contraction events may occur during the evolution process of *BrNRAMPs* and *BoNRAMPs*. In the course of hybridizing *B. rapa* with *B. oleracea* to create *B. napus*, the number of homologous genes corresponding to *AtNRAMP1/2/4/5/6* within the *B. napus* genome was comparable to the cumulative number of homologous genes to *AtNRAMP1/2/4/5/6* found in both *B. rapa* and *B. oleracea*. This observation serves to reassert the preservation and fidelity of the NRAMP gene family within Brassica species. However, phylogenetic analysis (Fig. 1) and chromosomal localization (Fig. 2) revealed that during evolution, the homologous gene originating from *BoNRAMP3.2* was lost in *B. napus*. This result demonstrated a moderate contraction of the NRAMP gene family in *B. napus* during this interspecies hybridization. The distinct contraction within the NRAMP gene family of Brassica species might be attributed to different selection pressures. Indeed, purifying selection occurred during the evolution (with Ka/Ks values consistently less than 1) of *B.napus*, indicating decreased selection pressure, which may ultimately lead to moderate contraction of *BnNRAMPs*.

Conserved motif and domain analysis aids in predicting protein functions and understanding the relationship between protein structure and function. The results indicate that motifs 1, 2, 5, and 7 could be pivotal for normal NRAMP protein function, as they are universally present. These motifs likely encompass the metal ion binding sites that are crucial for NRAMP functionality and are thus possibly retained throughout evolution. However, the location and composition of motifs 6 and 8 across the NRAMP family showed greater diversity. Subfamily I (NRAMP1/6) possesses motif 6, which is absent in subfamily II (NRAMP2/3/4/5). Additionally, except for NRAMP6s, all the other NRAMPs contain motif 8. Motif 8 is positioned centrally within the NRAMP2s/3s/4s/5s sequences, whereas it resides in the C-terminal region among the NRAMP1s. These findings highlight motif 6 and 8 as potential contributors to functional disparities within the NRAMP family, warranting further investigation into their role in the physiological functions of *NRAMPs*. Motifs 6 and 8, located within the transmembrane domain of NRAMP proteins, constitute a channel that facilitates the entry of divalent cations into cells in conjunction with motifs 1, 3, and 10 [36]. Therefore, variations in motifs 6 and 8 may lead to differences in the transport activity of these NRAMPs. Additionally, in the NRAMP of *Deinococcus radiodurans*, the substitution of threonine for histidine at position 230 results in a significant reduction in the uptake of Cd^2+^, while the uptake of Mn^2+^ and Fe^2+^ remains largely unaffected [37]. Therefore, motif 2 may be closely associated with the absorption of Cd ions.

The analysis of cis-regulatory elements is imperative for comprehending the mechanisms regulating gene expression and predicting gene functions. Most promoters of *BnNRAMPs* contain response elements for ABA, MeJA, GA, SA, or auxin, indicating potential hormonal regulation pathways. Studies have shown that plant hormones such as ABA, JA and SA are involved in plant responses to different metal stresses [38]. However, expression analysis revealed an overall subdued response of *BnNRAMPs* to these hormones and stress treatments. Similarly, research indicates that under exogenous ABA treatment, alterations in the *A. thaliana* NRAMP genes are not prominently observed [39]. Minimal changes in expression levels after hormone and stress treatments might be due to the fine-tuning of *NRAMP* gene expression and potential spatial regulation, altering NRAMP distribution without significant effects on overall expression levels. Additionally, the tissue-specific expression patterns of *BnNRAMPs* suggest that, apart from *BnNRAMP1.3/1.6/5.1/5.2*, the remaining *BnNRAMPs* do not exhibit noticeable tissue specificity. Similarly, the *Solanum tuberosum StNRAMP5* also shows tissue specificity [40]. However, *StNRAMP5* in *S. tuberosum* has a broader tissue specificity compared to *BnNRAMP5.1/5.2*, implying that *NRAMP5* may exert different functions in *B. napus* and *S. tuberosum*.

Apart from *those of BnNRAMP1.1/1.4/4.1/4.2*, the basal expression levels of the remaining *BnNRAMPs* are relatively low. Similar occurrences have been observed for species such as *Arachis hypogaea* L [33]. , *Solanum tuberosum* [40], *Phaseolus vulgaris* [41], and *Morus notabilis* [42], among others. Consequently, the relatively low expression levels of the NRAMP gene family may be a universal phenomenon. Notably, the comparatively low basal expression levels of *BnNRAMPs* might contribute to the minor changes observed in *BnNRAMPs* expression under the different hormonal and stress treatments mentioned above.

The expression patterns of *BnNRAMPs* in *B. napus* under Cd stress showed that *BnNRAMPs* had different response mechanisms to Cd. As the Cd concentration increased, there was a corresponding decrease in the expression levels of most *BnNRAMPs* (Fig. 8). Similar trends were observed in earlier investigations involving *Spirodela polyrhiza*. Compared to those in the control (0 h), the expression levels of *SpNRAMP1*, *SpNRAMP2*, and *SpNRAMP3* were downregulated at 6 h, 12 h, or 24 h of exposure to 50 µmol/L Cd [7]. However, in related *Glycine max* L. studies, an opposite trend was observed. The majority of *GmNRAMPs* (6 out of 10) showed upregulation, while a minority (3) exhibited downregulation [32]. This could be attributed to the use of higher Cd concentrations (100 µM) and shorter treatment durations (24 h) in soybean compared to other species. Alternatively, it may signify inherent expression differences among *NRAMPs* across different species. In the present study, the expression levels of *BnNRAMP6.1/6.2* also decreased under Cd treatment, but contrary to the general trend, a marginal increase in expression was noted in *BnNRAMP6.1/6.2* at higher Cd concentrations (50 µmol/L) compared to those at lower concentrations (25 µmol/L). This finding suggested a potential differential regulatory mechanism for *BnNRAMP6.1/6.2* under heightened stress conditions. In prior *B. napus* studies, the expression of *BnNRAMP6b* significantly increased after 4 h of exposure to 80 µmol/L Cd stress [43]. Similarly, under Cd stress treatment for 6–24 h, the expression levels of the NRAMP gene family in potato leaves exhibited an initial decrease followed by an increase [40]. Furthermore, within this investigation, *BnNRAMP5.1/5.2* exhibited a divergent response mechanism under Cd stress compared to that of other *BnNRAMPs*. *BnNRAMP5.1/5.2* expression increased at lower Cd concentrations (25 µmol/L). This suggests that *BnNRAMP5.1/5.2* is more sensitive to lower Cd levels, leading to increased expression in environments with low ionic strength. However, at a higher Cd concentration (50 µmol/L), *B. napus* seedlings might have experienced significant physiological toxicity, resulting in decreased expression of *BnNRAMP5.1/5.2*. However, a different scenario emerges in rice, where OsNRAMP5 serves as the primary transporter for cadmium uptake and transport [24, 25, 44]. The expression of *OsNRAMP5* decreases at lower Cd concentrations (10 µmol/L) [17]. This discrepancy may stem from varying sensitivities to Cd among different species, with rapeseed exhibiting greater cadmium tolerance compared to rice. These findings suggest intricate regulatory mechanisms governing *BnNRAMPs* expression under Cd stress, emphasizing the necessity for further exploration of the specific molecular responses of these transporters in *B. napus* under diverse stress conditions.

It is important to acknowledge the limitations of this study. While we conducted a comprehensive analysis of the NRAMP family at the bioinformatics level, this paper did not involve extensive experimental validation. As a result, many conclusions may lack robust support. Future research efforts can focus on identifying beneficial allelic variants of NRAMP for agriculture. For instance, the rice *OsNRAMP5-Q337K* mutant has shown the ability to accumulate less Cd while obtaining sufficient Mn [45], which holds significant implications for the development of low-cadmium-accumulating rice varieties applicable to agricultural production. Additionally, analysis of cis-regulatory elements has revealed probable regulation of the NRAMP gene family by various plant hormones, underscoring the importance of elucidating the intricate regulatory mechanisms imposed by these hormones. Furthermore, in addition to the NRAMP family, other families, such as zinc-regulated transporter-like proteins (ZIPs), heavy metal ATPases (HMA transporters), and metal tolerance or transporter proteins (MTPs), play crucial roles in mediating the absorption, transportation, and chelation of metal ions, notably Cd, within plant systems [42]. The synergistic interactions among these transporter proteins within plants highlight the imperative need to comprehend the intricate mechanisms governing their interactions. It is noteworthy that it is preferable to conduct research on NRAMP genes within the native species rather than solely focusing on the function of this gene in *A. thaliana*. This is because NRAMP may have different functions in different species. For example, rice OsNRAMP4 has been reported to be involved in the transport of Al ions, but there is no corresponding evidence in *A. thaliana*. We have successfully generated *NRAMP* gene knockout mutants in *B. napus*. Looking ahead, we anticipate shedding light on the specific role of the *NRAMP* gene in the absorption and transport of metal ions, particularly cadmium ions. This holds substantial importance in the context of developing rapeseed varieties with lower cadmium accumulation.

## Conclusions

This study involved a thorough exploration of the NRAMP gene family across Brassica species at the genome level. The investigation identified 37 *NRAMP* genes, 18 of which were in *B. napus*, 9 in *B. rapa*, and 10 in *B. oleracea*; these genes were classified into two subfamilies. Computational collinearity analysis suggested that these genes might have originated from either whole-genome duplication or segmental duplication events. All NRAMP proteins are hydrophobic, with the majority characterized as stable proteins. Subfamily I exhibited alkaline traits, while subfamily II exhibited acidic properties. Conservative motif analysis highlighted motif 6 and motif 8 as the probable primary contributors to the divergence between the two subfamilies. Subcellular localization assays indicated that, except for the potential vacuole localization of BrNRAMP1.2, the remaining NRAMP proteins were predominantly localized on the plasma membrane. Evolutionary and chromosomal analyses suggested that contraction occurred within the NRAMP gene family during the evolutionary progression of *B. napus*. The majority of NRAMP gene family members exhibited negligible tissue specificity across various tissues of *B. napus*. The cis-acting element analysis suggests that the NRAMP gene family in *B. napus* may be regulated by plant hormones, especially ABA and MeJA. Transcriptomic expression analysis indicates that hormones such as ABA, MeJA, and BL have an inductive effect on the expression of *BnNRAMP4.1/4.2*. However, with increasing treatment time, the expression levels of *BnNRAMP4.1/4.2* decrease. Under Cd treatment, expression analysis of *B. napus* reveals that the expression of most *BnNRAMP* genes may be negatively regulated by Cd, while *BnNRAMP5.1/5.2* and *BnNRAMP6.1/6.2* may have a complex regulatory mechanism distinct from other NRAMP genes. This study presents a comprehensive genome-wide identification and analysis of the gene structure of the NRAMP gene family within the Brassica species. Additionally, it reveals the adverse regulatory impact of Cd ions on NRAMP gene expression, as evidenced by expression level analysis. The findings from this study carry substantial reference value for subsequent functional explorations within the NRAMP gene family.

## Materials and methods

### Plant materials and growth conditions

In this study, our aim was to investigate the impact of Cd on the germination stage of *B. napus* seedlings and the response of *NRAMP* genes to Cd during this period. Therefore, in designing the experiment, we referenced previous research methods on the germination stage of *B. napus* seedlings [46, 47]. We utilized seeds from the inbred line of the *B. napus* variety Zhongshuang 11 as the primary material. The seeds selected exhibited full grains and uniform texture and underwent a sterilization process involving treatment with 70% ethanol (1 min), followed by triple rinsing with distilled water. Subsequently, these sterilized seeds were placed within seed germination boxes (6.3 × 6.3 × 9 cm) layered with four sheets of filter paper, accommodating 50 seeds per box. The irrigation process involved the application of 10 ml of a CdCl_2_·5/2H_2_O solution at concentrations of 0 µmol/L, 25 µmol/L, or 50 µmol/L. The germination process commenced in darkness at a controlled environment of 23 °C with a relative humidity of 70% for an initial period of 2 days. A cultivation period of 5 days was maintained under the following specified light conditions: light intensity, 300 µmol·m^− 2^ s^− 1^; temperature, 25 °C during the day; temperature, 22 °C at night; photoperiod, 16 h light and 8 h dark; and relative humidity, 70% [48]. Each treatment was replicated three times to ensure reliability and reproducibility. Sampling was conducted on the 7th day of the experiment. Immediately upon collection, the samples were subjected to rapid freezing in liquid nitrogen and subsequently stored at -80 °C for subsequent analyses and experimentation.

### Identification and evolutionary analysis of the NRAMP gene family in Brassica species

The protein sequences belonging to the NRAMP gene family were obtained from the *Arabidopsis thaliana* genome database (https://www.arabidopsis.org/). Homologous protein sequence alignments were performed against three Brassica species genome databases: *Brassica napus* multi-omics information resource (BnIR) (https://yanglab.hzau.edu.cn/BnIR/genome_data), *Brassica. oleracea* genome database (http://brassicadb.cn/download_genome/Brassica_Genome_data/Braol_JZS_V2.0), and the *Brassica. rapa* genome database (http://brassicadb.cn/download_genome/Brassica_Genome_data/Brara_Chiifu_V3.5), employing an E-value threshold of < 1e-10. Initial selection of *NRAMP* gene candidates was based on sequence similarity. Subsequent validation of candidate gene protein sequences was accomplished by utilizing the InterPro database (https://www.ebi.ac.uk/interpro/) to retrieve the hidden Markov model (HMM) (PF01566) specific to the NRAMP gene family. The application of the HMM confirmed the identification of *NRAMP* genes specific to the Brassica species.

To elucidate the physicochemical properties of the NRAMP gene family proteins, the ProtParam tool (https://web.ExPASy.org/protparam/) was utilized for analysis. Multiple sequence alignment was performed for the identified *NRAMPs* within both the Brassica species and *A. thaliana NRAMP* gene sets using MUSCLE (https://www.ebi.ac.uk/Tools/msa/muscle/). Additionally, a maximum likelihood estimation-based phylogenetic tree was constructed utilizing IQ-Trees. The resulting tree underwent visual enhancement through the use of Evolview (https://www.evolgenius.info/evolview-v2/).

Subcellular localization prediction of NRAMP family proteins was conducted utilizing two online tools, WoLFPSORT (https://wolfpsort.hgc.jp/) and Cell-PLoc 2.0 (http://www.csbio.sjtu.edu.cn/bioinf/plant/).

### Chromosomal localization and collinearity analysis of the Brassica species NRAMP gene family

TBtools [49] was used to chromosomally map the *NRAMPs* by utilizing the gene location data sourced from the Brassica species gff3 annotation file.

Collinearity analysis between the intragenomic and intergeneric genomes of *B. napus* was carried out using MCScanX, delineating homologous gene pairs within the *B. napus* genome as well as across Brassica species genomes. For the analysis of nonsynonymous (Ka) to synonymous (Ks) substitution rates, the simple Ka/Ks calculator function within TBtools [49] was employed.

### Conserved motifs, domains, and gene structure analysis of the Brassica species NRAMP gene family

The MEME Suite (https://meme-suite.org/meme/) was used to analyse conserved motifs present within the NRAMP gene family. Information regarding conserved domains within the NRAMP gene family was sourced from the Conserved Domains Database (CDD) and Resources (https://www.ncbi.nlm.nih.gov/Structure/cdd/cdd.shtml). The visualization of gene structure within the NRAMP gene family was accomplished using TBtools [49]. Additionally, the examination of cis-acting elements within the NRAMP gene family was performed utilizing the online tool PlantCARE (https://bioinformatics.psb.ugent.be/webtools/plantcare/html/).

### Analysis of the expression patterns of *BnNRAMPs* based on transcriptome data

We retrieved transcriptome data pertaining to *B. napus* from the BnIR (https://yanglab.hzau.edu.cn/BnIR/expression_zs11), encompassing tissue specific, stress-responsive, and hormone-induced expression profiles. The visualization of *B. napus NRAMP* gene expression data was conducted using the heatmap function available in TBtools [49].

### Analysis of the expression patterns of *BnNRAMPs* under cadmium stress

Oligonucleotide primers targeting the *BnNRAMPs* were designed utilizing Primer Premier 5 (Additional file 4). Total RNA was isolated with the FlaPure Plant Total RNA Extraction Kit sourced from Genesand Biotech Co., Ltd., based in Beijing, China. Subsequently, first-strand cDNA synthesis was accomplished using All-In-One 5X RT MasterMix manufactured by Applied Biological Materials, Inc., located at V6V 2J5, Canada. Quantitative real-time polymerase chain reaction (qRT‒PCR) analysis was performed utilizing the Bio-Rad CFX96 touch real-time PCR system (Bio-Rad, Hercules, CA, USA). qPCR was conducted using BlasTaq™ 2X qPCR MasterMix (also provided by Applied Biological Materials, Inc.) at V6V 2J5, Canada. The qPCR protocol involved an initial denaturation step at 95 °C for 3 min, followed by 39 cycles of denaturation at 95 °C for 15 s and annealing/extension at 60 °C for 1 min. A melting curve analysis was generated over a temperature range of 65 to 95 °C. For normalization, *BnACTIN2* (NM_001315560.1, LOC106390277) was utilized as the internal control. Relative expression levels of the *BnNRAMP* genes were determined using the 2^−∆∆Ct^ method [50]. The data are presented as the mean ± standard error of the mean (SEM). Statistical analysis was performed using GraphPad Prism 9.0 software, employing one-way analysis of variance (ANOVA).

### Electronic supplementary material

Below is the link to the electronic supplementary material.

Supplementary Material 1

Supplementary Material 2

Supplementary Material 3

Supplementary Material 4

## Data Availability

The datasets supporting the conclusions of this article are included within the article and its additional files. In this study, genomic data and annotation files for Arabidopsis thaliana, Brassica napus, Brassica rapa, and Brassica oleracea were obtained from TAIR (https://www.arabidopsis.org/), BnIR (https://yanglab.hzau.edu.cn/BnIR/genome_data), BRAD (http://brassicadb.cn/download_genome/Brassica_Genome_data/Braol_JZS_V2.0), and BRAD (http://brassicadb.cn/download_genome/Brassica_Genome_data/Brara_Chiifu_V3.5). Public transcriptome data can be accessed from BnIR (https://yanglab.hzau.edu.cn/BnIR/expression_zs11). The Zhongshuang11 seeds used in this research were provided by the Crop Research Institute, Hunan Academy of Agricultural Sciences.

## Abbreviations

aa: Amino acid
ABA: Abscisic acid
Al: Aluminum
ANOVA: One-way analysis of variance
AREs: Anaerobic responsive elements
As: Arsenic
BL: Brassinolide
BnIR: Brassica napus multi-omics information resource
Ca: Calcium
Cd: Cadmium
CDD: Conserved Domains Database
CDS: Coding sequence
Cu: Copper
Fe: Iron
GA: Gibberellin
HMA: Heavy metal ATPases
HMM: Hidden Markov Model
IAA: Indole-3-acetic acid
kDa: KiloDalton
MeJA: Methyl Jasmonate
Mg: Magnesium
Mn: Manganese
MTPs: Metal tolerance or transporter proteins
MW: Molecular weight
MYB: Myeloblastosis
NRAMP: Natural resistance-associated macrophage proteins
PI: Isoelectric point
qRT‒PCR: Quantitative reverse transcription Polymerase Chain Reaction
SA: Salicylic Acid
SEM: Standard Error of the Mean
TAIR: The Arabidopsis Information Resource
TPM: Transcripts per kilobase of exon model per million mapped reads
ZIP: Zinc-regulated transporter-like proteins
Zn: Zinc

## References

[1] Zhang H, Zhu J, Gong Z, Zhu JK (2022). Abiotic stress responses in plants. Nat Rev Genet.

[2] Zhao Y, Wang J, Huang W, Zhang D, Wu J, Li B, Li M, Liu L, Yan M. Abscisic-acid-regulated responses to Alleviate Cadmium toxicity in plants. Plants (Basel) 2023;12(5). 10.3390/plants12051023 PMC10005406 36903884

[3] Li D, Xu X, Hu X, Liu Q, Wang Z, Zhang H, Wang H, Wei M, Wang H, Liu H (2015). Genome-wide analysis and Heavy Metal-Induced expression profiling of the HMA Gene Family in Populus trichocarpa. Front Plant Sci.

[4] Lu Q, Weng Y, You Y, Xu Q, Li H, Li Y, Liu H, Du S (2020). Inoculation with abscisic acid (ABA)-catabolizing bacteria can improve phytoextraction of heavy metal in contaminated soil. Environ Pollut.

[5] Zhang W, Wang Z, Song J, Yue S, Yang H. Cd2 + uptake inhibited by MhNCED3 from Malus hupehensis alleviates Cd-induced cell death. Environ Exp Bot. 2019;166.

[6] Baba H, Tsuneyama K, Yazaki M, Nagata K, Minamisaka T, Tsuda T, Nomoto K, Hayashi S, Miwa S, Nakajima T (2013). The liver in itai-itai disease (chronic cadmium poisoning): pathological features and metallothionein expression. Mod Pathol.

[7] Chen Y, Zhao X, Li G, Kumar S, Sun Z, Li Y, Guo W, Yang J, Hou H. Genome-wide identification of the Nramp Gene Family in Spirodela polyrhiza and expression analysis under cadmium stress. Int J Mol Sci. 2021;22(12). 10.3390/ijms22126414 PMC8232720 34203933

[8] Zhang J, Zhang M, Song H, Zhao J, Shabala S, Tian S, Yang X. A novel plasma membrane-based NRAMP transporter contributes to cd and zn hyperaccumulation in Sedum Alfredii Hance. Environ Exp Bot. 2020;176.

[9] Tiwari M, Sharma D, Dwivedi S, Singh M, Tripathi RD, Trivedi PK (2014). Expression in Arabidopsis and cellular localization reveal involvement of rice NRAMP, OsNRAMP1, in arsenic transport and tolerance. Plant Cell Environ.

[10] Wang T, Li Y, Fu Y, Xie H, Song S, Qiu M, Wen J, Chen M, Chen G, Tian Y (2019). Mutation at different sites of Metal Transporter Gene OsNramp5 affects cd Accumulation and related agronomic traits in Rice (Oryza sativa L). Front Plant Sci.

[11] Ihnatowicz A, Siwinska J, Meharg AA, Carey M, Koornneef M, Reymond M (2014). Conserved histidine of metal transporter AtNRAMP1 is crucial for optimal plant growth under manganese deficiency at chilling temperatures. New Phytol.

[12] Nevo Y, Nelson N (2006). The NRAMP family of metal-ion transporters. Biochim Biophys Acta.

[13] Curie C, Alonso JM, Jean MLE, Ecker JR, Briat J-F (2000). Involvement of NRAMP1 from Arabidopsis thaliana in iron transport. Biochem J.

[14] Thomine S, Wang R, Ward JM, Crawford NM, Schroeder JI (2000). Cadmium and iron transport by members of a plant metal transporter family in Arabidopsis with homology to Nramp genes. Proc Natl Acad Sci U S A.

[15] Cailliatte R, Schikora A, Briat JF, Mari S, Curie C (2010). High-affinity manganese uptake by the metal transporter NRAMP1 is essential for Arabidopsis growth in low manganese conditions. Plant Cell.

[16] Chang JD, Huang S, Yamaji N, Zhang W, Ma JF, Zhao FJ (2020). OsNRAMP1 transporter contributes to cadmium and manganese uptake in rice. Plant Cell Environ.

[17] Ishimaru Y, Takahashi R, Bashir K, Shimo H, Senoura T, Sugimoto K, Ono K, Yano M, Ishikawa S, Arao T (2012). Characterizing the role of rice NRAMP5 in Manganese, Iron and Cadmium Transport. Sci Rep.

[18] Tejada-Jimenez M, Castro-Rodriguez R, Kryvoruchko I, Lucas MM, Udvardi M, Imperial J, Gonzalez-Guerrero M (2015). Medicago truncatula natural resistance-associated macrophage Protein1 is required for iron uptake by rhizobia-infected nodule cells. Plant Physiol.

[19] Zhang W, Yue S, Song J, Xun M, Han M, Yang H (2020). MhNRAMP1 from Malus hupehensis exacerbates cell death by accelerating cd uptake in Tobacco and Apple Calli. Front Plant Sci.

[20] Takahashi R, Ishimaru Y, Nakanishi H, Nishizawa NK (2014). Role of the iron transporter OsNRAMP1 in cadmium uptake and accumulation in rice. Plant Signal Behav.

[21] Lanquar V, Lelievre F, Bolte S, Hames C, Alcon C, Neumann D, Vansuyt G, Curie C, Schroder A, Kramer U (2005). Mobilization of vacuolar iron by AtNRAMP3 and AtNRAMP4 is essential for seed germination on low iron. EMBO J.

[22] Lanquar V, Ramos MS, Lelievre F, Barbier-Brygoo H, Krieger-Liszkay A, Kramer U, Thomine S (2010). Export of vacuolar manganese by AtNRAMP3 and AtNRAMP4 is required for optimal photosynthesis and growth under manganese deficiency. Plant Physiol.

[23] Xia J, Yamaji N, Kasai T, Ma JF (2010). Plasma membrane-localized transporter for aluminum in rice. Proc Natl Acad Sci U S A.

[24] Chang JD, Huang S, Konishi N, Wang P, Chen J, Huang XY, Ma JF, Zhao FJ (2020). Overexpression of the manganese/cadmium transporter OsNRAMP5 reduces cadmium accumulation in rice grain. J Exp Bot.

[25] Tang L, Dong J, Qu M, Lv Q, Zhang L, Peng C, Hu Y, Li Y, Ji Z, Mao B (2022). Knockout of OsNRAMP5 enhances rice tolerance to cadmium toxicity in response to varying external cadmium concentrations via distinct mechanisms. Sci Total Environ.

[26] Ishikawa S, Ishimaru Y, Igura M, Kuramata M, Abe T, Senoura T, Hase Y, Arao T, Nishizawa NK, Nakanishi H (2012). Ion-beam irradiation, gene identification, and marker-assisted breeding in the development of low-cadmium rice. Proc Natl Acad Sci U S A.

[27] Chalhoub B, Denoeud F, Liu S, Parkin IA, Tang H, Wang X, Chiquet J, Belcram H, Tong C, Samans B (2014). Plant genetics. Early allopolyploid evolution in the post-neolithic Brassica napus oilseed genome. Science.

[28] Liu Z, Coulter JA, Li Y, Zhang X, Meng J, Zhang J, Liu Y (2020). Genome-wide identification and analysis of the Q-type C2H2 gene family in potato (Solanum tuberosum L). Int J Biol Macromol.

[29] Cailliatte R, Lapeyre B, Briat JF, Mari S, Curie C (2009). The NRAMP6 metal transporter contributes to cadmium toxicity. Biochem J.

[30] Maser P, Thomine S, Schroeder JI, Ward JM, Hirschi K, Sze H, Talke IN, Amtmann A, Maathuis FJ, Sanders D (2001). Phylogenetic relationships within cation transporter families of Arabidopsis. Plant Physiol.

[31] Mani A, Sankaranarayanan K (2018). In Silico Analysis of Natural Resistance-Associated macrophage protein (NRAMP) family of transporters in Rice. Protein J.

[32] Qin L, Han P, Chen L, Walk TC, Li Y, Hu X, Xie L, Liao H, Liao X (2017). Genome-wide identification and expression analysis of NRAMP Family genes in soybean (Glycine Max L). Front Plant Sci.

[33] Tan Z, Li J, Guan J, Wang C, Zhang Z, Shi G. Genome-wide identification and expression analysis reveals roles of the NRAMP gene family in Iron/Cadmium interactions in peanut. Int J Mol Sci 2023, 24(2). 10.3390/ijms24021713 PMC9866697 36675227

[34] Bian X, Cao Y, Zhi X, Ma N. Genome-wide identification and analysis of the Plant Cysteine Oxidase (PCO) Gene Family in Brassica napus and its role in abiotic stress response. Int J Mol Sci 2023, 24(14). 10.3390/ijms241411242 PMC10379087 37511002

[35] Cheng F, Wu J, Wang X (2014). Genome triplication drove the diversification of Brassica plants. Hortic Res.

[36] Bozzi AT, Gaudet R (2021). Molecular mechanism of Nramp-Family Transition Metal Transport. J Mol Biol.

[37] Bozzi AT, Bane LB, Weihofen WA, McCabe AL, Singharoy A, Chipot CJ, Schulten K, Gaudet R (2016). Conserved methionine dictates substrate preference in Nramp-family divalent metal transporters. Proc Natl Acad Sci U S A.

[38] Ma X, Yang H, Bu Y, Zhang Y, Sun N, Wu X, Jing Y (2023). Genome-wide identification of the NRAMP gene family in Populus trichocarpa and their function as heavy metal transporters. Ecotoxicol Environ Saf.

[39] Pan W, You Y, Shentu JL, Weng YN, Wang ST, Xu QR, Liu HJ, Du ST (2020). Abscisic acid (ABA)-importing transporter 1 (AIT1) contributes to the inhibition of cd accumulation via exogenous ABA application in Arabidopsis. J Hazard Mater.

[40] Tian W, He G, Qin L, Li D, Meng L, Huang Y, He T (2021). Genome-wide analysis of the NRAMP gene family in potato (Solanum tuberosum): identification, expression analysis and response to five heavy metals stress. Ecotoxicol Environ Saf.

[41] Ishida JK, Caldas DGG, Oliveira LR, Frederici GC, Leite LMP, Mui TS (2018). Genome-wide characterization of the NRAMP gene family in Phaseolus vulgaris provides insights into functional implications during common bean development. Genet Mol Biol.

[42] Fan W, Liu C, Cao B, Qin M, Long D, Xiang Z, Zhao A (2018). Genome-wide identification and characterization of four gene families putatively involved in Cadmium Uptake, translocation and sequestration in Mulberry. Front Plant Sci.

[43] Meng JG, Zhang XD, Tan SK, Zhao KX, Yang ZM (2017). Genome-wide identification of Cd-responsive NRAMP transporter genes and analyzing expression of NRAMP 1 mediated by miR167 in Brassica napus. Biometals.

[44] Yang M, Zhang Y, Zhang L, Hu J, Zhang X, Lu K, Dong H, Wang D, Zhao FJ, Huang CF (2014). OsNRAMP5 contributes to manganese translocation and distribution in rice shoots. J Exp Bot.

[45] Zhao FJ, Chang JD (2022). A weak allele of OsNRAMP5 for safer rice. J Exp Bot.

[46] Zhou T, Yue CP, Zhang TY, Liu Y, Huang JY, Hua YP (2021). Integrated ionomic and transcriptomic dissection reveals the core transporter genes responsive to varying cadmium abundances in allotetraploid rapeseed. BMC Plant Biol.

[47] Zhou ZS, Song JB, Yang ZM (2012). Genome-wide identification of Brassica napus microRNAs and their targets in response to cadmium. J Exp Bot.

[48] Zhang F, Xiao X, Wu X (2020). Physiological and molecular mechanism of cadmium (cd) tolerance at initial growth stage in rapeseed (Brassica napus L). Ecotoxicol Environ Saf.

[49] Chen C, Wu Y, Li J, Wang X, Zeng Z, Xu J, Liu Y, Feng J, Chen H, He Y (2023). TBtools-II: a one for all, all for one bioinformatics platform for biological big-data mining. Mol Plant.

[50] Livak KJ, Schmittgen TD (2001). Analysis of relative gene expression data using real-time quantitative PCR and the 2(-Delta Delta C(T)) method. Methods.

