# Supplementary material for: Genome-wide identification and evolutionary analysis of the NRAMP gene family in the AC genomes of Brassica species
Source: BMC Plant Biol. 2024 Apr 23;24:311. doi: 10.1186/s12870-024-04981-1 (PMC11036763; doi:10.1186/s12870-024-04981-1)
Supplement: Supplementary file 4 — Supplementary Material 4 [file 12870_2024_4981_MOESM4_ESM.docx]

**Additional file 4.** The sequences of primers for qPCR

| Primer | Primer Sequence 5’ to 3’ |
| --- | --- |
| *BnNRAMP1.1/1.4* | F:CGCACAACCTCTTCCT |
|  | R:GCAAACAGCACCACTTA |
| *BnNRAMP1.2/1.5* | F:ATGCGAGGATTTGGAC |
|  | R:TTAGGAAGTTTCTGAGCC |
| *BnNRAMP1.3/1.6* | F:ACTCTGCGTTGGTTCTC |
|  | R:TTTAGCTCGGTCTTCG |
| *BnNRAMP2.1/2.4* | F:TTGGAGTTACGGTCTGC |
|  | R:GGAAGTTCAATGGAGGAG |
| *BnNRAMP2.2/2.3* | F:GAGGTCATCGGTAGTGC |
|  | R:AAAGACAAACCCATAGTTG |
| *BnNRAMP3.1/3.2* | F:TGATGAGCATTGCGTTTC |
|  | R:GAGCCTCGCCGATAAG |
| *BnNRAMP4.1/4.2* | F:TTTGGGCTGGTGTTGT |
|  | R:AGTTCCACTGGGTTTCG |
| *BnNRAMP5.1/5.2* | F:AGTCCAGGAAGACCAATC |
|  | R:AACCGCTGTCACAAAGA |
| *BnNRAMP6.1/6.2* | F:GCCGTCGTCTTCTTAG |
|  | R:AGTGTATCCGCTGTCTG |
| *BnACTIN2* | F:CTGGATTCTGGTGATGGT |
|  | R:GCTTCTCCTTGATGTCTCT |
